# Attitudes of Palestinian Health-Care Professionals in Gaza to Clinical Practice Guideline for Diagnosis and Treatment of Diabetes Mellitus

**DOI:** 10.3389/fendo.2017.00288

**Published:** 2017-10-31

**Authors:** Mahmoud Radwan, Ali Akbari Sari, Arash Rashidian, Amirhossein Takian, Sanaa Abou-Dagga, Aymen Elsous

**Affiliations:** ^1^Department of Health Management and Economics, School of Public Health, International Campus, Tehran University of Medical Sciences (IC-TUMS), Tehran, Iran; ^2^International Cooperation Directorate, Palestinian Ministry of Health, Gaza City, Palestine; ^3^Health Equity Research Center (HERC), Tehran University of Medical Sciences (TUMS), Tehran, Iran; ^4^Department of Research Affairs and Graduates Studies, Islamic University of Gaza, Gaza City, Palestine

**Keywords:** barriers, adherence, clinical practice guideline, diabetes mellitus, psychometric properties

## Abstract

**Background:**

Despite the huge numbers of the internationally produced and implemented Clinical Practice Guidelines (CPGs), the compliance with them is still low in health care. This study aimed at assessing the attitudes of Palestinian health-care professionals toward the most perceived factors influencing the adherence to the CPG for Diabetes Mellitus in the Primary Health-care centers of the Ministry of Health (PHC-MoH) and the Primary Health-care centers of the United Nations Relief and Works Agency for Palestine Refugees (PHC-UNRWA) using a validated questionnaire.

**Methods:**

A cross-sectional design was employed with a census sample of all Palestinian family doctors and nurses (*n* = 323). The Cabana theoretical framework was used to develop a study questionnaire. A cross cultural adaptation framework was followed to develop the Arabic version questionnaire. The psychometric properties of Arabic version were finally assessed.

**Results:**

The Arabic version questionnaire showed a good construct validity and internal consistency reliability. The overall adherence level to the diabetic guideline was disappointingly suboptimal 51.5% (47.3% in the PHC-MoH and 55.5% in the PHC-UNRWA) *P* = 0.000. The most frequently perceived barriers in the PHC-MoH were lack of incentives, lack of resources, and lack of guideline trustworthiness, whereas the lack of time and the lack of guideline trustworthiness were the most prominent barriers in the PHC-UNRWA. In spite of the lack of trustworthiness of the diabetic guideline, most respondents in both settings had a positive attitude toward guidelines in general, but this attitude was not a predictor of guideline adherence.

**Conclusion:**

The good validity and reliability of our questionnaire can provide support for the accuracy of our findings. Multifaceted implementation strategies targeting the main barriers elicited from this study are required for addressing the lack of incentives, organizational resources, lack of confidence in the guideline, and time constraints.

## Introduction

Diabetes Mellitus (DM) is a serious chronic disease and an increasingly important public health issue. It is a major cause of blindness, kidney failure, heart attacks, stroke, and lower limb amputation (1). The World Health Organization (WHO) estimates that, worldwide, about 422 million people aged over 18 years have diabetes in 2014 with a global prevalence of 8.5% among adult population (1). The highest prevalence rate (13.7%) of DM is in the WHO-Eastern Mediterranean Region (1). It was the eighth leading cause of death among both sexes in 2012 (1). In Palestine, it has been projected that the prevalence of DM among Palestinians will be approximately 23.4% in 2030 (2). The primary health-care services in Palestine are delivered by two main providers; the Ministry of Health (MoH) and the United Nations Relief and Works Agency for Palestine Refugees in the Near East (UNRWA). Both of the MoH and UNRWA are structurally, functionally, and financially separated and provide an extensive range of community health services. The UNRWA provides its services to Palestinian refugees only whereas the MoH is responsible to provide its services to refugees and non-refugees. The refugees patients receive free of charge services from the UMRWA, while the patients who seek the care in the MoH should be insured and pay the cost sharing. On the other hand, the UNRWA personnel receive salaries of 1.5 times higher than in the MoH. Unlike the MoH, the UNRWA has its own regular budget, which has contributed in continuing the availability of medical supplies (e.g., medicines, laboratory consumables, and medical equipment). Moreover, the UNRWA has its own systematic training programs while the MoH often relies on donor projects who can secure the funds for training activities and provide most of training materials. The daily average number of patient seen by physicians in the UNRWA was 82 in 2016 (3) and estimated to be 48 in MoH. In the Gaza Strip, the diabetic patients receive their health care through 49 clinics in the PHC-MoH and 22 clinics in the PHC-UNRWA. The Palestinian CPG for DM was adapted from international guidelines and targeted the areas of screening, diagnosis, and treatment in order to standardize the care provided to patients with Type 1 and Type 2 diabetes (4).

Clinical Practice Guidelines (CPGs) are “systematically developed statements to assist practitioners’ and patients’ decisions about appropriate health care for specific clinical circumstances” (5). The interest in the CPGs as an important knowledge translation tools has been increasing in the past decade (6). They are recognized as tools for advancement of evidence based medicine, are useful tools for improving the quality of services, can improve patient outcomes, and contain the costs by decreasing unnecessary variations in care (7). In spite of the huge numbers of the internationally produced and implemented CPGs, the compliance with them is still low among health-care workers (8, 9). Several studies revealed that the CPGs achieved moderate results in changing the process of care (10, 11). In USA, only about 55% of the patients received the care based on the CPG recommendations (12). Such poor adherence to CPGs does not merely lead to provision of suboptimal care, but it can threaten patient safety, waste resources, and create poor health outcomes (13). Despite the great endeavors to translate guidelines into clinical practice, their implementation is a complicated process influenced by many variables such as professional’s behavior, the guidelines themselves, and the method of implementing the recommendations (14). A systematic review of 76 published study included 120 survey conducted by Cabana and his colleagues on barriers to physician guideline adherence in relation to behavior change (14). They developed a framework, which comprises of seven general barriers that are classified into three main categories: knowledge related barriers (lack of familiarity and lack of awareness); attitude-related barriers (lack of agreement, lack of self-efficacy, lack of outcome expectancy, and lack of motivation/inertia of previous practice); and behavior-related barriers (patient factors, guideline factors, and environment factors). The appropriate analysis of the factors hindering the health-care professionals from being able to practically implement the CPGs is the main initial step toward enhancing the adherence to CPGs (15). This study aimed at exploring the adherence level and the most perceived barriers of the adherence to the CPG for DM in both the PHC-MoH and PHC-UNRWA using a validated questionnaire.

## Materials and Methods

### Study Design and Sampling

A cross-sectional design was employed with a census sample of all Palestinian family doctors and nurses (*n* = 323) who worked with chronic patients in 71 PHCc (49 in the MoH and 22 in the UNRWA) in Gaza Strip. The total number of eligible doctors and nurses working in the PHC-MoH was 124 and 51, respectively, while the total number of eligible doctors and nurses working in the PHC-UNRWA was 115 and 56, respectively. All those working doctors and nurses with at least 1 year working experience were included.

### Questionnaire Development

Based on the Cabana Framework (14), this questionnaire was designed after reviewing the previous relevant questionnaires. Most of the relevant items measuring the constructs of Cabana framework were adapted and considered. Specific items of the dimensions (organizational constraints, lack of resources, and lack of reimbursements) were tailored to match with the local context. A preliminary questionnaire with 54 items (11 dimensions) to assess the barriers of adherence to CPG for DM and 10 items to assess the demographic and work background were included. Another 10 key recommendations derived from the Palestinian CPG for DM were included to measure the adherence level. A 5-point Likert scale was used for response categories with the rating scale of “strongly agree,” “agree,” “neither agree nor disagree,” “disagree,” and “strongly disagree.”

### Translation and Validation of the Questionnaire

**Figure 1:**
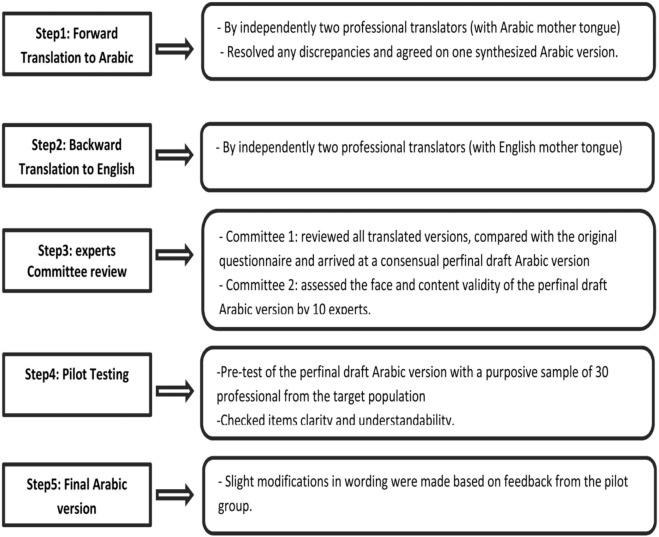
The steps of questionnaire translation.

The guideline of cross cultural adaptation process was recruited in translation of the questionnaire (16) where the detailed steps are described in Figure 1. In order to check face and content validity, the final draft Arabic questionnaire was independently validated by 10 experts (academics, health experts, endocrinologists, and family doctors). Content Validity Index was calculated to rate the relevance of the questionnaire items (17). All items were rated as relevant with scores over 0.87. Slight editorial changes in the wording and the structure of the key recommendations items were made based on the consensus among the author (MR), two endocrinologists, and one family doctor. Finally, the questionnaire was piloted among 30 of the eligible participants. The results of the pilot study revealed a good overall Cronbach’s alphas of 0.85, while the internal consistency of the domains ranged between 0.60 and 0.91.

### Data Collection

From June 2016 to August 2016, data were gathered by four data collectors after one full day of training about the study scope and objectives, questionnaire items, and the potential areas for misconception. The face-to-face interview-based questionnaire was used as a method of data collection. We obtained three formal approvals for data collection; from the MoH, UNRWA, and the Palestinian health research council in Gaza. This council was formed by the MoH and, after 2012, it has been officially delegated as an independent council with full responsibility to ensure keeping of all research ethical standards. Oral approvals were taken from all respondents before participation in this study.

### Data Analysis

Data analysis was carried out with SPSS version 20. The codes of the negative worded questions were reversed before data analysis. Descriptive statistics were used to describe the sample characteristics. Categorical variables were described using frequencies and percentages, whereas means and SDs were used to represent continuous data. *P*-values of 0.05 or less were considered significant. Psychometric properties of the study questionnaire were assessed by.

#### Construct Validity (Factor Analysis)

An exploratory factor analysis was conducted to explore the construct and underlying factor structure of the questionnaire. Extraction was performed using Principal Components Analysis (PCA) with Varimax rotation (18). Kaiser normalization was used to assess the appropriateness for factor analysis and sample adequacy. Outcomes of the exploratory factor analysis were considered accurate if the Kaiser–Meyer–Olkin (KMO) measure of sampling adequacy of ≥0.7, and Bartlett’s test of Sphericity with a *P*-value of <0.001 (19). The Kaiser Criterion with eigenvalues of ≥1 (20) was used to identify the number of extracted factors. Items with a factor loading of more than 0.40 were included in the retained factors. Item loading is an important indicator during factor analysis, which indicates of the correlation strength of each retained item to the underlying factor (domain).

#### Internal Consistency Reliability

Internal consistency reliability of the questionnaire was assessed by calculating Cronbach’s alpha coefficient of the overall questionnaire and the identified domains where score over than 0.70 were considered good, reflecting the internal correlation between items of the same area (21). Since the questionnaire encompasses many negatively worded items, reverse coding was conducted to make sure that a higher score always means a more positive response.

## Results

### Psychometric Properties of the Questionnaire

#### Factor Analysis

In this study, the sample size was quite adequate for factor analysis as the KMO measure was 0.90. Such a high value can yield reliable factors (22). Bartlett’s test of Sphericity demonstrated that the inter-item correlations were highly significant (χ^2^ = 15,166.2; df = 1,431; *P* < 0.001). The PCA analysis indicated a total of 10 factors with eigenvalues of ≥1, which accounted for 73.04% of the variance (Table 1). The new factors structure seems to be highly reasonable and reflects a more conceptual construct over this data set.

**Table 1:** Exploratory factor analysis of overall questionnaire (*n* = 53).

Factors	Item number	Item loading	Eigenvalues	Explained variance, %	Cronbach α
F1—agreement	Q32, Q33, Q34, Q35, Q36, Q37, Q38, Q39, Q40, Q41, Q42	0.845, −0.784, −779, 0.719, 0.859, −0.858, 0.837, 0.835, −755, 0.853, 0.824	14.34	26.55	0.95
F2—knowledge and skills	Q22, Q24, Q25, Q26, Q27, Q28, Q29, Q30, Q31, 43, Q45	0.719, 0.671, −0.808, 0.723, 0.614, 0.735, 0.858, 0.593, 0.634, −0.780, −0.805	10.02	18.55	0.93
F3—lack of recourses	Q23, Q57, Q58, Q59, Q60, Q61, Q62	0.647, 0.735, 0.752, −0.583, −0.853, −0.856, −0.680	3.53	6.55	0.92
F4—motivation/Inertia of previous practice	Q44, Q46, Q47, Q48, Q49, Q50	0.497, 0.590, 0.781, 0.758, 0.815, 0.526	2.48	4.60	0.88
F5—lack of time	Q51, Q52, Q53, Q54	0.834, 0.843, 0.835, 0.860	2.03	3.77	0.91
F6—patients factors	Q72, Q73, Q74, Q75	0.805, 0.775, 0.787, 0.702	1.78	3.29	0.83
F7—lack of incentives	Q63, Q64, Q65, Q66	0.479, 0.800, 0.779, 0.683	1.56	2.90	0.88
F8—guideline trustworthiness	Q70, Q71	0.857, 0.884	1.37	2.54	0.90
F9—organizational support	Q55, Q56	0.702, 0.695	1.20	2.23	0.92
F10—guideline clarity	Q67, Q68, Q69	−0.504, 0.595, 0.581	1.09	2.02	0.62

Overall				73.04	0.93

*Extraction Method: principal component analysis*.

*Rotation Method: Varimax with Kaiser Normalization*.

#### Internal Consistency Reliability

After conducting the construct validation, the Cronbach’s alpha was computed (Table 1). The overall Cronbach’s alpha was 0.93, which indicates a high correlation and consistency between the items and the questionnaire. The internal consistency of the new factors ranged between 0.83 and 0.95 and considered very good except one factor with α Cronbach 0.68.

### Respondent Characteristics

Of the 346 eligible participants, 323 responded to the interview based questionnaires yielding a response rate of 93.3%. The respondent characteristics are summarized in Table 2.

**Table 2:** Respondent characteristics (*n* = 323).

		PHC-MoH (*n* = 159)		PHC-UNRWA (*n* = 164)		Overall *N* = 323	
**Attributes**		***n***	**%**	***n***	**%**	***N***	**%**

Sex	Male	97	61.0	93	56.7	190	58.8
	Female	62	39.0	71	43.3	133	41.2

Age	<35	38	23.9	58	35.4	96	29.7
	35–44	70	44.0	64	39.0	134	41.5
	45–60	51	32.1	42	25.6	93	28.8
	M (SD)	40.70 (7.91)		39.38 (8.32)		40.03 (8.13)	

Qualification	Diploma	11	6.9	19	11.6	30	9.3
	Bachelor	119	74.8	118	72.0	237	73.4
	Postgrad	29	18.3	27	16.4	56	17.3

Specialization	Medicine	114	71.7	111	67.7	225	69.7
	Nursing	45	28.3	53	32.3	98	30.3

Position	Practitioner	126	79.2	152	92.7	278	86.1
	Manager	33	20.8	12	7.3	32	9.9

Total work experience	≤5 years	34	21.4	42	25.6	76	23.5
	6–10 years	38	23.9	49	29.9	87	26.9
	11–20 years	63	39.6	42	25.6	105	32.5
	>20 years	24	15.1	31	18.9	55	17.0
	M (SD)	12.96 (8.00)		12.27 (10.00)		12.6 1 (8.35)	

Current work experience	≤5 years	57	35.8	80	48.8	137	42.4
	6–10 years	58	36.5	46	28.0	104	32.2
	≥11 years	44	27.7	38	23.2	82	25.4
	M (SD)	8.42 (5.53)		8.33 (6.69)		8.37 (6.14)	

### Adherence to Diabetic Guideline

Table 3 shows that the overall adherence mean score across the key recommendations was (51.5%, SD = 7.3). Significantly higher total adherence was found in the PHC-UNRWA (55.5%, SD = 5.7) compared to the PHC-MoH (47.3%, SD = 6.3) *P* = 0.000. A small proportion of the respondents 11.5% (1.3% in PHC-MoH and 21.3% in PHC-UNRWA) claimed that they were always or often adherents to implementing the key recommendations. The vast majority 84.2% (89.9% in PHC-MoH and 78.7% in PHC-UNRWA) claimed that they were sometimes adherents to implementing the key recommendations. The least adherence was on the recommendation to perform screening for type 2 DM in all individuals at age of ≥45 years old (0.6% in PHC-MoH and 2.4% in PHC-UNRWA) followed by the prescription of angiotensin-converting-enzyme inhibitor to normotensive type 2 patients if urine albumin/creatinine ratio is positive 3 months later (1.3% in PHC-MoH and 7.3% in PHC-UNRWA).

**Table 3:** Adherence level to the diabetic guideline.

Key recommendations	PHC-MoH (*n* = 159)				PHC-UNRWA (*n* = 164)			
	Always often	Sometimes	Rarely never	Don’t know	Always often	Sometimes	Rarely never	Don’t know

	*n* (%)	*n* (%)	*n* (%)	*n* (%)	*n* (%)	*n* (%)	*n* (%)	*n* (%)
1. Drs. request HbA1c every 3–4 months for all diabetic pts. with unstable glycemic control	1 (0.6)	49 (30.8)	109 (68.6)	0 (0.0)	4 (2.4)	81 (49.4)	79 (48.2)	0 (0.0)
2. Drs. work to achieve FPG of 90–130 mg/dl and 2-h post-PPG of 140–180 mg/dl	101 (63.5)	53 (33.3)	5 (3.1)	0 (0.0)	150 (91.5)	13 (7.9)	1 (0.6)	0 (0.0)
3. Doctors work to achieve a reduction in the BP for adult diabetic Pts. below 130/80 mmHg	107 (67.3)	47 (29.6)	5 (3.1)	0 (0.0)	144 (87.8)	19 (11.6)	1 (0.6)	0 (0.0)
4. Drs. work to achieve a total serum cholesterol for diabetic Pts. of 200–220 mg/dl	102 (64.2)	56 (35.2)	1 (0.6)	0 (0.0)	145 (88.4)	19 (11.6)	0 (0.0)	0 (0.0)
5. For newly diabetic Pts. type 2, Drs start therapy with education, diet, and exercise for 2–4 months	24 (15.1)	92 (57.9)	43 (27.0)	0 (0.0)	84 (51.2)	78(47.6)	2(1.2)	0 (0.0)
6. Screening for type 2 diabetes mellitus (DM) is performed in all individuals at age of ≥45 years old	1 (0.6)	1 (0.6)	157 (98.8)	0 (0.0)	4 (2.4)	7 (4.3)	153 (93.3)	0 (0.0)
7. Drs confirm the Dx of DM if result of first FPG and the repeating result ≥126 mg/dl	111 (69.8)	46 (28.9)	2 (1.3)	0 (0.0)	149 (90.9)	14 (8.5)	1 (0.6)	0 (0.0)
8. Drs. prescribe Statin to diabetic Pts. if high LDL + poor response of dietary mange, exercise	9 (5.7)	79 (49.7)	71 (44.7)	0 (0.0)	61 (37.2)	84 (51.2)	19 (11.6)	0 (0.0)
9. Fundoscopy for type 1 & 2 DM is performed yearly and more often if retinopathy is progressing	69 (43.4)	67 (42.1)	23 (14.5)	0 (0.0)	111 (67.7)	47 (28.7)	6 (3.7)	0 (0.0)
10. Drs. prescribe ACE to normotensive type 2 Pts. if urine Alb./Creat. is positive 3 months later	2 (1.3)	9 (5.7)	140 (88.1)	8 (5.0)	12 (7.3)	66 (40.2)	81 (49.4)	5 (3.0)
*n* (%)	2 (1.3)	143 (89.9)	14 (8.8)	0 (0.0)	35 (21.3)	129 (78.7)	0 (0.0)	0 (0.0)
Overall percentage	Always often (11.5%)			Sometimes (84.2%)		Rarely never (4.3%)		
Mean (SD)	47.3 (6.3)				55.5 (5.7)			

Overall	Mean = 51.5, median = 51.6, SD = 7.3							

### The Perceived Barriers of Adherence to the Diabetic Guideline

Table 4 shows the following results.

**Table 4:** Perceived Barriers of adherence to the diabetic guideline.

Barriers		Work setting	5&4	3	2&1	*N*	Mean	SD	*t*	*P*-value
				
			*n* (%)	*n* (%)	*n* (%)					
1	Knowledge and skills	PHC-MoH	71 (44.7)	78 (49.1)	10 (6.2)	159	58.68	14.00	−10.872	0.000
		PHC-UNRWA	140 (85.4)	23 (14.0)	1 (0.6)	164	75.46	13.73		
2	Agreement	PHC-MoH	163 (85.6)	17 (10.6)	6 (3.8)	159	79.45	16.57	0.665	0.507
		PHC-UNRWA	132 (80.5)	27 (16.5)	5 (3.0)	164	78.17	17.99		
3	Motivation/inertia of previous practice	PHC-MoH	137 (86.2)	19 (11.9)	3 (1.9)	159	75.01	14.93	−4.528	0.000
		PHC-UNRWA	153 (93.3)	10 (6.1)	1 (0.6)	164	82.13	13.31		
4	Lack of time	PHC-MoH	28 (17.0)	68 (41.5)	68 (41.5)	159	40.88	17.59	−3.481	0.001
		PHC-UNRWA	10 (6.3)	56 (35.2)	93 (58.5)	164	36.68	14.17		
5	Organizational constraints	PHC-MoH	13 (8.2)	86 (54.1)	60 (37.7)	159	37.29	13.62	−8.760	0.000
		PHC-UNRWA	72 (43.9)	73 (44.5)	19 (11.6)	164	56.46	24.10		
6	Lack of resources	PHC-MoH	7 (4.4)	42 (26.4)	110 (69.2)	159	34.64	11.53	−25.227	0.000
		PHC-UNRWA	163 (82.9)	23 (14.0)	5 (3.1)	164	70.52	13.87		
7	Lack of incentives	PHC-MoH	18 (11.3)	32 (20.1)	109 (68.6)	159	33.86	14.86	−9.818	0.000
		PHC-UNRWA	64 (39)	70 (42.7)	30 (18.3)	164	52.89	19.57		
8	Guideline trustworthiness	PHC-MoH	15 (9.5)	60 (37.7)	84 (52.8)	159	34.96	12.05	−5.524	0.000
		PHC-UNRWA	38 (23.1)	79 (48.2)	47 (28.7)	164	43.10	14.29		
9	Guideline clarity	PHC-MoH	29 (18.3)	95 (59.7)	35 (22.0)	159	46.28	14.47	−4.913	0.000
		PHC-UNRWA	55 (33.6)	97 (59.1)	12 (7.3)	164	55.20	17.89		
10	Patient factor	PHC-MoH	145 (91.2)	13 (8.2)	1 (0.6)	159	77.54	13.33	0.705	0.481
		PHC-UNRWA	150 (91.4)	12 (7.3)	2 (1.3)	164	76.46	14.24		

	Adherence	PHC-MoH				159	47.35	6.30	−12.156	0.000
		PHC-UNRWA				164	55.54	5.79		

*5, 4, 3, 2, and 1 indicate respondents strongly agree, agree, are neutral, disagree, and strongly disagree, respectively*.

#### Knowledge and Skills

The mean score of the knowledge and skills was 67.2%, SD = 16.1. Significantly higher total knowledge and skills was found in the PHC-UNRWA (75.4%, SD = 13.7) compared to the PHC-MoH (58.6%, SD = 14.0) *P* = 0.000. Compared to 44.7% in the PHC-MoH, the vast majority of doctors and nurses (85.4%) in the PHC-UNRWA claimed that they had the adequate knowledge and skills to implement the recommendations of the diabetic guideline.

#### Agreement

The mean score of the professionals attitude toward the guidelines in general was 78.8%, SD = 17.2. There were insignificant differences in attitude toward the CPGs among respondents working in the PHC-MoH (79.4%, SD = 16.5) and the PHC-UNRWA (78.1%, SD = 17.9) *P* = 0.507. Most of the PHC-MoH participants (88%) and PHC-UNRWA participants (85.4%) agreed that the CPGs are good educational tools. The vast majority of PHC-MoH participants (90.6%) and the PHC-UNRWA participants (88.4%) agreed that implementing the diabetic guideline recommendations lead to improvement in the quality of health care.

#### Motivation/Inertia of Previous Practice

The mean score of the motivation/inertia of previous practice was 78.6%, SD = 14.5. Most of respondents in the PHC-UNRWA (82.1%, SD = 13.3) were more likely to be motivated than in the PHC-MoH (75.0%, SD = 14.9) *P* = 0.000. More than 93% and 86% of respondents in the PHC-UNRWA and the PHC-MoH, respectively, reported that they were enthusiastic to comply with implementing the diabetic guideline and they were able to cope with the change toward working under standardized instructions.

#### Lack of Time

The mean score of the lack of time was 37.8%, SD = 16.2. There was significant variation in perceiving the lack of time among doctors and nurses working in the PHC-MoH (34.6%, SD = 14.1) and the PHC-UNRWA (40.8%, SD = 17.5) *P* = 0.001.

#### Organizational Constraints

The mean score of the organizational constraints was 47.0%, SD = 21.8. Doctors and nurses in the PHC-MoH (37.2%, SD = 13.6) were more likely to perceive the organizational constraints than in the PHC-UNRWA (56.4%, SD = 24.1) *P* = 0.000. Only 8.3% of the participants in the PHC-MoH compared to 43.9% in the PHC-UNRWA stated that the top management is committed with supporting the implementation of diabetic guideline and the job description facilitates its implementation.

#### Lack of Resources

The mean score of the lack of resources was 52.8%, SD = 22.0. Significantly wide variation in perceiving the lack of resources was shown between the PHC-MoH (34.6%, SD = 11.5) and the PHC-UNRWA (70.5%, SD = 13.8) *P* = 0.000.

#### Lack of Incentives

The mean score of the lack of incentives was 43.5%, SD = 19.8. More than half of the respondents in the PHC-UNRWA (52.8%, SD = 19.5) agreed more favorably with the current incentives than those in the PHC MoH (33.8%, SD = 14.8) *P* = 0.000.

#### Guideline Trustworthiness

The mean score of the professionals perception toward the trustworthiness of diabetic guideline was 39.1%, SD = 13.8. Perception of guideline trustworthiness yielded a statistically significant difference (*P* = 0.000) among participants in the PHC-MoH (34.9%, SD = 12.0) and in the PHC-UNRWA (43.1%, SD = 14.2).

#### Guideline Clarity

The mean score of the guideline clarity was 50.8%, SD = 16.8. Significantly higher score of the guideline clarity was revealed in the PHC-UNRWA (55.2%, SD = 17.8) compared to the PHC-MoH (46.2%, SD = 14.4) *P* = 0.000. Compared to 33.6% of the respondents in the PHC-UNRWA, only 18.3% in the PHC-MoH claimed that the layout of the diabetic guideline was well-coordinated and its key recommendations were specific and unambiguous.

#### Patient Factor

The mean score of the patient factor was 76.9%, SD = 13.7. Statistically insignificant variation (*P* = 0.481) in perceiving the patient factor was shown between the PHC-MoH (77.5%, SD = 13.3) and the PHC-UNRWA (76.4%, SD = 14.2). The overwhelming majority of doctors and nurses in the PHC-MoH (91.2%) and the PHC-UNRWA (91.4%) agreed that the diabetic patients wanted doctors to comply with the diabetic guideline and the patient’s preferences were consistent with its recommendations.

## Discussion

We developed and tested a questionnaire assessing the barriers of adherence to the CPG for DM. The analysis showed very good psychometric properties. It had a good construct validity and internal consistency reliability. To the best of our knowledge, this is the first study to assess the factors hindering the adherence to the CPG for DM in the Palestinian PHC-MoH and PHC-UNRWA by using a valid and reliable questionnaire based on a previously popular framework (14). The overall adherence level was suboptimal (51.5%). In Indonesia, the adherence to the recommendations of diabetic guideline was low and varied between 2 and 45% (23). In West Bank/Palestine, only 21.0% of the professionals widely implemented the diabetic guideline (24), whereas, in Egypt, 43.3% of the family physicians were appropriately adherent to diabetic neuropathy guideline (25). Generally, the adherence to CPGs is moderate (14) or even low (8). Many systematic reviews pointed out that most of the adherence enhancing interventions had only modest to moderate effects (6, 26). Such limited effects might be due to the improper use of the behavioral and organizational theories as a guide for enhancing the adherence (27). Therefore, we employed a popular theoretical framework and went through the analysis of the barrier factors as an initial step toward understanding the professionals’ perspectives and enhancing the adherence interventions (15). Overall, our study showed that there were many significant differences in perceiving the barriers and adherence among the health-care professionals in the PHC-MoH and PHC-UNRWA except for the attitude/agreement and patient factor. We can initially exclude the attitudinal barrier toward the guideline adherence since doctors and nurses in the PHC-MoH and the PHC-UNRWA had a similarly quite positive attitude toward the CPGs (28). Most of the PHC-MoH participants (88%) and PHC-UNRWA participants (85.4%) agreed that the CPGs are good educational tools (29). The vast majority of PHC-MoH participants (90.6%) and the PHC-UNRWA participants (88.4%) agreed that implementing the diabetic guideline recommendations lead to improvement in the quality of health care (28). A possible explanation for this positive attitude is that the overwhelming vast majority of the participants in both settings agreed that the diabetic patients wanted doctors to comply with the diabetic guideline and the patient’s preferences were consistent with its recommendations. However, such attitude can favorably be invested to as a key potential factor in the implementation of CPGs.

The better adherence to the diabetic guideline in the PHC-UNRWA compared to the PHC-MoH may reflect the higher levels of awareness and familiarity in the UNRWA settings. In comparison with (85.9%) of the PHC-UNRWA doctors and nurses, only 27.1% of the PHC-MoH doctors and nurses were aware about the availability of the diabetic guideline copies. In West Bank/Palestine, only 35.9% of professionals had a copy of the diabetic guideline (24), while in Estonia, 76% of doctors had a copy of the diabetic guideline available (30). The dominant minority (6.3%) of the PHC-MoH had an easy access to the guideline at any time compared to (82.3%) in the PHC-UNRWA. The relatively poor knowledge in the PHC-MoH is more likely due to the poor dissemination strategy. The inadequate production and dissemination of the guidelines might cause the unfamiliarity with them (31). Previous studies indicated that knowledge is a key factor for a greater adherence to the diabetic CPGs (32, 33); nonetheless, high awareness about the diabetic guideline does not necessarily guarantee the adherence to its recommendations (23). Although the appropriate knowledge and positive attitude are indispensable, but they do not guarantee the guideline adherence (14). However, the poor adherence suggests that the health-care professionals seem to be confronted with the external related barriers rather than the attitude or knowledge factors.

The most frequently perceived barriers in the PHC-MoH were the lack of incentives, lack of resources, and lack of the guideline trustworthiness, whereas the lack of time and the lack of the guideline trustworthiness were the most prominent barriers in the PHC-UNRWA. The motivational incentives were much less mentioned by the professionals in the PHC-MoH compared to the professionals in the PHC-UNRWA. The lack of incentives was identified as a barrier for implementing the diabetic guideline (34). Our study revealed that the overwhelming majority of PHC-MoH respondents (92.4%) stated that the current monthly salary, the encouragement by work colleagues (88.6%), and the acknowledgment by line and senior management (80.5%) did not motivate them to comply with the diabetic guideline. In the absence of conclusive evidence on the impact of financial incentives on the quality of diabetic management, a recently systematic review concluded that the pay for performance have variable impacts on physician behavior (35). Another study to assess the impact of financial incentives on providers’ adherence to evidence-based smoking cessation practice guidelines revealed that financial incentives alone did not result in recommendations adherence (36). It seems sensible to analyze what could motivate the health-care professionals prior any guideline implementation. Hence, the Palestinian national payment method and the incentive scheme should be carefully reviewed and redesigned, taking into account the monetary and non-monetary incentives.

The lack of resources at the PHC-MoH played a main role in impeding the adherence to the diabetic guideline. Availability of resources such as lab equipment and medications has been identified as a main contributor to implementing the diabetic guideline (33). Similarly, all participants identified inadequacy of resources as the main barrier to the implementation of the national stroke CPGs (37). The MoH essential drugs and medical disposables lists include about 480 item of drugs and more than 900 item of medical disposables. Mostly, more than 40% of these needed items are either completely unavailable or in critically low stocks (38). This chronic lack of essential drugs and disposables could largely impede the proper adherence to the guideline recommendations and consequently pose a very serious threat to the patient treatment regimen. A logic explanation for the difference in the adherence to the recommendations of diabetic guideline (e.g., doing HbA1c) between the MoH and UNRWA is the lack of resources. Although the lack of resources is extremely challenging in the Palestinian context, the decision makers are strongly invited to judge the most efficient ways for rational use of the scarce resources (6).

The lack of the guideline trustworthiness as the third perceived barrier affected the professionals both in the PHC-MoH and PHC-UNRWA. Lack of agreement with the guideline recommendations due to less trust in them and their inapplicability was perceived as the most prominent barrier among Dutch general practitioners (39). Our analysis showed that all of the PHC-MoH respondents (100%) and (95.1%) of the PHC-UNRWA respondents claimed that the diabetic guideline had not been developed based on rigorous evidences, in the same time, 83% of the PHC-MoH respondents and 63.4% of the PHC-UNRWA respondents claimed that it had not been developed by professional experts. Someone could argue this finding and suggest interpretation as that the diabetic guideline has not been sold well to the health professionals. However, our finding is robustly supported by a recent study aimed at assessing the methodological quality of the current diabetic guideline using the AGREE II instrument, which revealed that the largest domain of “Rigor of Development” had a weak score (40). Therefore, it is crucial for guideline developers to consider the systematic approach in synthesizing the evidences and selecting the recommendations. Using the published Arabic version of the AGREE II instrument as a valid appraisal tool is strongly recommended for developing, adopting, adapting, or updating any future guideline.

The most common barrier cited by the PHC-UNRWA respondents was the lack of time. The massive majority of doctors and nurses (93.7%) in the PHC-UNRWA claimed that implementing the diabetic guideline adds extra efforts over their essential assigned tasks. More than 91% of them reduced the consultation time with diabetic patients due to the heavy workload and 93% were unable to adhere to the guideline recommendations due to the large numbers of outpatient visitors. Time constraints and pressure of work have been found to be main challenges to the guideline implementation (33). It could be suggested to analyze the workloads prior the implementation of guidelines or at least ensure that the available numbers of professionals are adequate.

### Strengths and Limitations

The evaluation of the barriers to the CPG for DM was totally based on a common and widely used theoretical framework by Cabana et al. (14). Many steps were taken to assess the validity and reliability of the developed questionnaire and this provide support for the trustworthiness of our findings. The adherence was assessed based on 10 key recommendations elicited from the exiting diabetic guideline in order to ensure a common understanding among participants and achieve a maximum representation of the various main recommendations. A potential limitation of our study is the reliance on self-reported data, which may led to recall bias and social desirability bias. In spite of such limitation, there is evidence revealed that self-reporting is a valid and reliable source for assessing the physician’s performance since its findings are quite congruent with the findings of the medical records (41).

## Conclusion

The results of our analysis give considerable support to the Cabana theoretical framework, as a model for assessing the barriers and enablers to the CPGs. Our study shows that the adherence to CPG for DM in the PHC-UNRWA is a bit higher than in the PHC-MoH, and the perceived barriers among doctors and nurses in the PHC-MoH were higher than their counterparts in the PHC-UNRWA. The most perceived barriers among doctors and nurses in the PHC-MoH were lack of incentives, lack of resources, and lack of the guideline trustworthiness, while the lack of time was the most eminent barrier in the PHC-UNRWA followed by lack of the guideline trustworthiness. Despite the generally positive attitude toward guidelines among doctors and nurses in both PHC-MoH and PHC-UNRWA, it was not a predictor of guideline adherence (29). The knowledge was higher among the professionals in the PHC-UNRWA than in the PHC-MoH, and it appears to be a predictor of guideline adherence. Multifaceted implementation strategies targeting the main barriers elicited from this study are extremely required for addressing the incentives, organizational resources, the rigor of guideline development, and time constraints. Recent evidence concluded that a tailored implementation strategy targeting perceived barriers is useful for improving the guideline adherence (42). Further qualitative studies to allow a better and deep understanding of the factors influencing the appropriate adherence of the CPGs are widely encouraged.

## Author Contributions

All authors have contributed significantly in this research work. The authors (MR, AS, AR, AT) significantly contributed in the study design and the critical review of the manuscript. The principal investigator (MR) collected, analyzed, interpreted the data, and wrote the first draft of the manuscript. The authors (SA-D, AE) remarkably contributed in the analysis and interpretation of data. Final approval was given by all authors.

## Conflict of Interest Statement

The authors declare that the research was conducted in the absence of any commercial or financial relationships that could be construed as a potential conflict of interest.
